# Evaluation of Olive Leaf Phenolic Compounds’ Gastrointestinal Stability Based on Co-Administration and Microencapsulation with Non-Digestible Carbohydrates

**DOI:** 10.3390/nu16010093

**Published:** 2023-12-27

**Authors:** Carmen Duque-Soto, Francisco Javier Leyva-Jiménez, Rosa Quirantes-Piné, María Asunción López-Bascón, Jesús Lozano-Sánchez, Isabel Borrás-Linares

**Affiliations:** 1 Department of Food Science and Nutrition, Faculty of Farmacy, University of Granada, Campus Universitario Cartuja s/n, 18071 Granada, Spain; carmenduque@ugr.es; 2 Area of Food Science and Technology, Faculty of Chemical Sciences and Technologies, University of Castilla-La Mancha, Avda. Camilo José Cela 10, 13071 Ciudad Real, Spain; javier.leyva@uclm.es; 3 Regional Institute for Applied Scientific Research (IRICA), University of Castilla-La Mancha, Avda. Camilo José Cela 10, 13071 Ciudad Real, Spain; 4 Department of Analytical Chemistry, Faculty of Sciences, University of Granada, Avda Fuentenueva s/n, 18071 Granada, Spain; iborras@ugr.es; 5 Research and Development Functional Food Centre (CIDAF), Health Science Technological Park, Avenida del Conocimiento 37, Edificio BioRegión, 18016 Granada, Spain; alopez@cidaf.es

**Keywords:** olive leaf extract, phenolic compounds, microencapsulation, spray-drying, in vitro gastrointestinal digestion, bioaccessibility

## Abstract

The large generation of olive by-products has motivated their revalorization into high-added-value products. In this regard, olive leaves pose as an interesting source of bioactive compounds, due to their phenolic content with commonly known antioxidant, anti-inflammatory, and immunomodulatory properties, with potential application in non-communicable diseases. However, their effectiveness and applicability into functional foods is limited by their instability under gastrointestinal conditions. Thus, the development of protective formulations is essential. In this study, the spray-drying encapsulation of a phenolic-rich olive leaf extract with inulin as the encapsulating agent was optimized. Then, the behavior of the free extract under gastrointestinal conditions, its co-administration with the encapsulating agent, and the optimized microencapsulated formulation were studied through an in vitro gastrointestinal digestion process following the INFOGEST protocol. Digestion of the free extract resulted in the degradation of most compounds, whereas this was minimized in the co-administration of the non-encapsulated extract with the encapsulating agent. This protective effect, related to its interaction with inulin, was similar to the microencapsulated formulation. Thus, both approaches, co-administration and microencapsulation with inulin, could be promising strategies for the improvement of the stability of these anti-inflammatory and immunomodulatory compounds under gastrointestinal conditions, enhancing their beneficial effect.

## 1. Introduction

Food industry by-products have emerged as promising sources of bioactive compounds and have been proposed for their revalorization into the production of high-added-value products, promoting a circular economy approach [1,2]. In this regard, the olive tree is one of the main crops cultivated in Mediterranean countries, which hold 97% of global production, increasing exponentially each year [3,4]. With Spain being the country with the highest production, waste accumulation associated with the production of olive oil is high, implying the production of 1–5 t/ha of pruning residue, including leaves and branches [3,4,5]. Its abundance and accumulation have been supposed to be an environmental problem, as they have been traditionally ground and burned, increasing contaminant CO_2_ emissions. Thus, a strategy for environmental remediation has emerged through their revalorization, associated with their interesting composition with regards to phytochemicals, especially phenolic compounds, and reducing the production cost through monetizing food waste [6,7].

In this regard, phenolic compounds have gained interest throughout the scientific community for their functional and technological properties, which make them interesting compounds for the development of functional foods. Specifically, phenolic-rich extracts from different products derived from the olive tree have shown great antioxidant [8,9], anti-inflammatory [10,11,12], immunomodulatory [13], anti-hypertensive [14], hypocholesterolemic [15], hypoglycemic [16], and cardioprotective effects [17]. This bioactivity is especially significant for their impact on the treatment and prevention of non-communicable diseases, such as obesity [18], diabetes [19], and inflammatory intestinal diseases [20,21,22], pathologies whose incidence has increased dramatically during the last decades. More specifically, hydroxytyrosol and oleuropein, the most abundant phenolic compounds in olive leaves, have shown an anti-inflammatory effect on intestinal conditions, such as ulcerative colitis [23,24,25]. Additionally, these health benefits have been related to the impact of these compounds on the colonic microbiota, modulating gut microbial composition, promoting probiotic bacteria while inhibiting pathogenic strains [26,27].

However, their bioactive potential is dependent on their gastrointestinal release from the matrix, intestinal absorption, and pharmacokinetic behavior. In fact, their bioactivity, and consequently health benefits, may be limited by their instability under gastrointestinal conditions. Specifically, phenolic compounds present poor water solubility and stability, being sensitive to unfavorable environments such as high temperatures, light, oxygen, low pH, and enzymatic activity, with most of these occurring under gastrointestinal conditions, which renders them as non-stable substances during digestion [28,29,30]. In fact, oleuropein, among other phenolic compounds from olive structures, has been shown to be heavily degraded under gastrointestinal conditions, being affected by the low pH values found in the fasting gastric conditions [31,32,33]. Additionally, they are rapidly metabolized to polar compounds and eliminated from the body, mainly in the urine [34]. Hence, the nature of their oral administration could compromise their observed effect through a reduction in their bioaccessibility, as the gastrointestinal conditions may enhance this degradation.

To solve this problem, the encapsulation of these bioactive compounds has been proposed as a useful strategy to ensure their protection throughout the gastrointestinal tract while allowing for a controlled release in the areas of interest, modulating their access to absorption sites and interaction with colonic microbiota and enhancing their effect on human health. However, the encapsulation technique and conditions applied are limited by the physicochemical stability of phenolic compounds. In this sense, spray-drying has been widely used for bioactive molecules, due to its low cost and industrial scalability [35,36]. This methodology also presents a number of advantages for bioactive compound encapsulation, including a reduced exposure to high temperatures, high quality and stability of the resulting microcapsules, and a low operating cost [37,38]. Among the possible encapsulating agents, non-digestible carbohydrates present an incredible opportunity for colon-targeted delivery of compounds. In this sense, inulin, a fructan-type non-digestible polysaccharide, can be catabolized by the colonic microbiota, thus allowing for a transportation of these compounds to their desired sites of action and their interaction with colonic microbiota [39,40,41]. Additionally, previous studies have established the bioactive potential of this polysaccharide, as it has proved to be an outstanding prebiotic agent, regulating blood sugar and lipids, as well as possessing antioxidant, anticancer, and immune regulation activities, among others [42].

Prior to their implementation, the behavior of the resulting microcapsules under biological conditions needs to be assessed to evaluate their transportation ability. While both in vivo and in vitro models are available, due to the complexity and cost of the former, the latter have emerged as desirable alternatives in the accurate representation of the digestive process. The use of in vitro gastrointestinal digestion protocols supposes an approach to a better understanding of the behavior of phenolic compounds under these conditions, as well as allowing consideration of the protective effect that microencapsulation may bring to their phenolic profile. In this sense, the INFOGEST protocol constitutes a harmonized and standardized protocol which allows a correct representation of the gastrointestinal tract as well as a comparison between studies [43,44]. However, although encapsulation of olive leaf extracts has been reported in the literature, the comparison of the phenolic profile resulting from the digestion of these encapsulated formulations with the non-encapsulated phenolic compounds co-administered with inulin is yet to be assessed [37]. In this sense, with this co-administration, the protective effect due to the interaction of inulin with phenolic compounds on their stability under gastrointestinal conditions could be evaluated. Indeed, this comparative evaluation would be essential for an improvement in the understanding of the digestion of phenolic compounds and their protection from gastrointestinal conditions, as well as to improve the development of functional foods with scalable and effective protective strategies.

Therefore, the aim of this study was the evaluation of the differential phenolic profiles, under in vitro gastrointestinal conditions, of a microencapsulated, free co-administered formulation with inulin and free olive leaf phenolic-rich extract in order to improve protection under gastrointestinal conditions. Thus, the optimization of the microencapsulation of olive leaf extract with inulin using spray-drying was performed using the response surface methodology (RSM). Then, the optimum microencapsulated formulation together with the free formulations (non-encapsulated olive leaf phenolic extract and its combined administration with inulin) were assayed under gastrointestinal conditions to study the potential improvement/enhancement in colon bioaccessibility and the protection of the phytochemicals from the olive leaf extract. The in vitro gastrointestinal digestion was carried out following the INFOGEST protocol for the evaluation of the effect of both co-administration with inulin and the encapsulation strategy by spray-drying on its protection.

## 2. Materials and Methods

### 2.1. Chemicals

Commercial olive leaf extract was kindly provided by Deretil S.L. (Cuevas del Almanzora, Spain), enriched in hydroxytyrosol and oleuropein. For the encapsulation processes, inulin was purchased from Fagron (Barcelona, Spain). Ethanol and LC-MS-grade methanol and acetonitrile was acquired from Fisher Chemicals (Waltham, MA, USA), whereas acetic and formic acids were from Sigma-Aldrich (Steinheim, Germany). Milli-Q water was purified using a Milli-Q system (Millipore, Bedford, MA, USA). Moreover, hydroxytyrosol, oleuropein, luteolin-7-O-glucoside, and loganin standards were acquired from Sigma-Aldrich or Extrasynthese (Genay Cedex, France). For in vitro digestion, enzymes (pepsin 3412 U/mg and pancreatin 4xUSP) and bovine bile salts (Sigma B-8631) were purchased from Sigma-Aldrich (Saint Louis, MO, USA). Chemicals for the preparation of simulated digestive fluids, hydrochloric acid (HCl), sodium hydroxide (NaOH), sodium chloride (NaCl), potassium dihydrogen phosphate (KH_2_PO_4_), potassium chloride (KCl), sodium hydrogen carbonate (NaHCO_3_), and ammonium carbonate ([(NH_4_)_2_CO_3_]), were purchased from Fisher Chemicals (Waltham, MA, USA).

### 2.2. Microencapsulation of the Olive Leaf Phenolic-Rich Extract by Spray-Drying

The optimization of the microencapsulation of olive leaf extract with inulin by spray-drying was performed using the response surface methodology (RSM) based on a central composite design (CCD) 2^2^ model with a star and four central points (Statgraphics Centurion version XVI supported by Statpoint Technologies, Warrenton, VA, USA). The effect of the independent variables, specifically, air temperature (134.75–195.25 °C) and extract:inulin ratio (0.68–4.315), on the response variables, the encapsulation efficiencies (%EE) of hydroxytyrosol (HT) and oleuropein (OLE), was evaluated. The experimental results were fitted to a second-order polynomial model, as shown in Equation (1):

(1) $$Y=\beta 0+\sum_{\;i=1}^{n}\beta_{i}X_{i}+\sum_{i=1\;}^{n}\beta_{ii}\;X_{i}^{2}+\sum_{i=1}^{n}\sum_{j=i+1}^{n}\beta_{ij}\;X_{i}\;X_{j}$$

where *Y* represents the response variable; *β*_0_ is the response constant coefficient fixed at the central point of the experiments; *β_i_*, *β_ii_*, and *β_j_* are the regression coefficients of the linear, quadratic, and interaction terms, respectively; and *X_i_* and *X_j_* represent the values of the independent variables. With the purpose of evaluating the adequacy of the proposed model and the adjustment of the obtained data, three different parameters were assessed (model adequacy, coefficient of determination (R^2^), and lack-of-fit test). Moreover, optimization was performed using the desirability function on those responses with higher fitting. Moreover, the desirability function is an approach that enables the identification of the simultaneous optimum conditions, estimating the global desirability of the responses (the best condition for both responses) in each run. For that purpose, the desirability function takes values from 0 to 1, where values close to 1 reveal the best conditions to achieve the proposed optimization [45]. All experimental runs were carried out randomly in a spray-dryer 4M8-TriX instrument (ProCept, Zalzate, Belgium) comprising a process column, an angled T transport tube, a cyclone, and a product manifold.

For the encapsulation process, inulin (in a range of 0.69–4.32 g) was previously dissolved in water (in quantities between 48.31 and 44.68 g) at 70 °C until obtaining a homogeneous solution. After that, 1 g of the extract was added and mixed by stirring until complete dilution (50 g), obtaining a total feeding solids from 3.37% to 10.63%. The drying procedures were carried out setting the conditions as follows: inlet air temperature, 135–195 °C; airflow, 0.30 m^3^/min; feeding flow, 2 mL/min; atomization air flow, 13 L/min; nozzle diameter, 0.6 mm; and differential pressure of cyclone, 15–16 mbar. Whereas the outlet air temperature was maintained in the range of 60–90 °C. The attained microparticles after each procedure were kept protected from light and moisture at room temperature.

### 2.3. Encapsulation Efficiency (%EE) Assesment

In order to determine the encapsulation efficiency, the phenolic contents of both the non-encapsulated and encapsulated fractions were calculated. First, to recover the non-encapsulated fraction, 150 mg of microparticles was added in 1 mL of MeOH-EtOH, 50:50 (*v*/*v*), and dispersed using gentle agitation. The obtained suspension was centrifuged at 90× *g* at 4 °C for 1 min, recovering the supernatant which was later centrifuged at 360× *g* at 4 °C for 1 min. Finally, the obtained supernatant was filtered through a 0.2 µm PTFE filter and maintained at −20 °C and protected from light exposure for later analysis. For the extraction of the encapsulated compounds, 150 mg of microparticles was added to 0.75 mL of MeOH-EtOH, 50:50 (*v*/*v*), and vortex-mixed for 1 min. Then, the microparticles were introduced into a refrigerated ultrasound bath for 20 min and centrifuged at 15,000× *g* at 4 °C for 10 min. The procedure was repeated on the obtained pellet and both supernatants were combined, centrifuged at 15,000× *g* at 4 °C for 10 min, filtered through a 0.2 µm cellulose filter, and maintained at −20 °C and protected from light for later analysis. The EE% was determined using Equation (2), described elsewhere:

(2) $$EE\%=\frac{Total\;compound\;content-Non\;encapsulated\;compound\;content}{Total\;compound\;content}\times 100$$

### 2.4. INFOGEST Static In Vitro Digestion

Olive leaf extract, in the free co-administered formulation with inulin and its microencapsulated form with inulin as the encapsulating agent, was subjected to static in vitro gastrointestinal digestion following the harmonized INFOGEST protocol described in Minekus et al., 2014 with modifications proposed by Brodkorb et al., 2018 regarding phenolic compounds [43,44]. The digestions were carried out in triplicate for each sample: commercial olive leaf extract (5 g), olive leaf extract combined with inulin (1.3 g of extract with 3.7 g of inulin), and olive leaf extract microencapsulated with inulin as the encapsulating agent (5 g).

For the simulation of oral digestion, 5 g of each substrate was dissolved in 5 mL (1:1, *w*/*w*) of simulated salivary fluid (SSF) in a 50 mL centrifuge tube, protected from light exposure, and vortexed for 5 min.

Then, 7.5 mL of simulated gastric fluid (SGF) containing 2000 U/mL of pepsin, and 5 µL of 0.3 M CaCl_2_ was added to the resulting bolus. The pH was adjusted to 3.0 by addition of 1 M aqueous HCl and milli-Q H_2_O was added until a final volume of 18 mL was achieved. The resulting mixture was homogenized, inertized with N_2_ flow to ensure anaerobic conditions, and incubated for 120 min at 37 °C under constant agitation at 150 rpm using a thermostatic incubator (MaxQTM 6000 SHKE6000-8CE, Thermo Scientific, Waltham, MA, USA). Thereafter, samples (1 mL) were taken at the end of the gastric digestion and stored at −80 °C for their later analysis.

Subsequently, to stop gastric digestion, the pH was increased to 7.0 by adding aqueous 1 M NaOH. For the intestinal phase, 9.8 mL of simulated intestinal fluid (SIF), 100 U/mL of pancreatin, 2.5 mL of bile, 40 µL of 0.3 M CaCl_2_ and, finally, Milli-Q H_2_O was added to achieve a final volume of 40 mL. The resulting mixture was homogenized and inertized with N_2_ as previously mentioned. Thus, the intestinal phase was carried out for 2 h, maintaining conditions of temperature and agitation of 37 °C and 150 rpm (MaxQTM 6000 SHKE6000-8CE, Thermo Scientific, Waltham, MA, USA). Then, samples (1 mL) were taken at 30 min intervals and stored at −80 °C for their later analysis.

Throughout the in vitro digestion process, pH measurements were obtained at intervals of 30 min, adjusting the value to pH 3.0 in the gastric phase and pH 7.0 in the intestinal phase with 1 M aqueous HCl or NaOH when necessary.

### 2.5. Bioactive Compound Extraction

Before phenolic characterization of the digestates by HPLC-MS, the digested samples were defrosted on ice for 2 h for those stored in microcentrifuge tubes or overnight in the refrigerator for those in centrifuge tubes. The samples from all digestive phases were homogenized and centrifuged at 19,500× *g*, at 4 °C for 10 min, separating both supernatants (bioaccessible fraction) and pellets (residual fractions).

The extraction of phenolic compounds was performed as previously described [46]. For evaluation of the bioaccessible fraction, 100 µL of MeOH-EtOH, 50:50 (*v*/*v*), was added to 200 µL of the supernatant, vortexed, and stored at −20 °C for 30 min for protein precipitation. Then, samples were centrifuged at 19,500× *g*, at 4 °C for 10 min and the supernatants evaporated in a vacuum concentrator (Eppendorf Concentrator plus) at ambient temperature for 4–5 h. Dried residues were re-suspended in 100 µL of MeOH before characterization, homogenized in a refrigerated ultrasound bath, and centrifuged at 19,500× *g*, at 4 °C for 10 min, introducing the supernatants in HPLC vials for their analysis.

As for the residual fractions, 1 mL of MeOH was added to 100 mg of residue, homogenized, and introduced into a refrigerated ultrasound bath for 15 min. Then, it was agitated at 4 °C and centrifuged at the previous described conditions. The samples were introduced into the vacuum concentrator at ambient temperature for 2–3 h and stored at −20 °C until analysis. Previous to the analysis, the samples were re-suspended in MeOH to a concentration of 500 µg/mL. The centrifuged supernatants were also introduced into HPLC vials for their later analysis.

### 2.6. Bioactive Compound Bioaccessibility

The bioaccessibility was calculated as previously described [46] using Equation (3) [47], corresponding to the phenolic compound fraction freed from the studied formulation into the gastrointestinal tract at the end of the simulation (240 min of the intestinal phase) and, thus, accessible for intestinal absorption. Additionally, the accumulative presence throughout the digestion process of the bioaccessible fraction was calculated using Equation (4), as a percentage of the initial composition of the extract [48]. The initial phenolic content in the olive leaf extract for the bioaccessibility evaluation was assessed by re-suspending 5 g of extract in the final volume of the intestinal phase (18 mL) prior to being submitted to the extraction procedure described in the previous section.

(3) $$Bioaccesibility\left(\%\right)=\frac{PC\;content\;in\;IP4\;(mg)}{Initial\;PC\;content\;(mg)}\times 100\%$$

(4) $$Recovery\left(\%\right)=\frac{PC\;content\;in\;DS\;(mg)}{Initial\;PC\;content\;\left(mg\right)}\times 100\%$$

where PC is phenolic compounds; IP4 is the final sample from the intestinal phase (time 240 min); DS is the digested samples for each phase; and initial PC content is the phenolic content present in the olive leaf extract.

### 2.7. Bioactive Compound Characterization Using HPLC-MS

Analyses were performed using an Agilent 1200 liquid chromatography system (Agilent Technologies, Palo Alto, CA, USA) equipped with a micro vacuum degasser, binary pump, autosampler, thermostatic column compartment, and diode array detector. The HPLC column used for separation was an Agilent Zorbax Eclipse Plus C18 (1.8 μm, 4.6 × 150 mm). The mobile phases consisted of water plus 0.1% formic acid (A) and acetonitrile (B). The multi-step linear gradient applied was the following: 0 min, 5% B; 2 min, 30% B; 25 min, 95% B; 30 min, 95% B; 32 min, 5% B and then, the initial conditions were maintained for 3 min. The flow was 0.5 mL/min, the temperature was maintained fixed at 25 °C, and the injection volume in the HPLC system was 5 μL.

The HPLC system was coupled to a microTOF mass spectrometer (Bruker Daltoniks, Bremen, Germany) equipped with an ESI interface (Agilent Technologies, Palo Alto, CA, USA) operating in negative-ion mode, in a mass range of 50–1000 *m*/*z*. Nitrogen was used as a nebulizing/ionizing and drying gas at conditions of 2 bar and 10 L/min. The drying temperature was set at 190 °C, capillary voltage of +4 kV, and end-plate offset at −500 V. Other optimum values for the ion-transfer parameters were output voltage, 120 V; skimmer 1, 40 V; hexapole 1, 23 V; hexapole RF, 100 Vpp; skimmer 2, 22.5 V; lens 1 transfer, 50 µs; and lens 1 pre-pulse storage, 3 µs.

In order to recalibrate mass spectra obtained during analysis to achieve a mass precision of 5 ppm, 5 mM sodium formate was use as calibration agent at the beginning of each analysis.

### 2.8. Data Processing

The chemical characterization of the phenolic compounds in the free extract, the microencapsulated formulation, and the digested samples (bioaccessible and residual fractions) was carried out using the software DataAnalysis 4.0 (Bruker Daltoniks, Bremen, Germany). For identification purposes, the mass analyzer data (exact mass and isotopic pattern) were processed for obtaining a molecular formulae list of the analyzed compounds with a 5 ppm tolerance error. The elucidation was achieved by comparing the obtained putative molecular formulae with the previous literature and personal databases of phenolic compounds present in this plant matrix.

In addition, for the quantification of the olive leaf extract, microparticles and the different digestate samples, the injection into the HPLC-TOF-MS instrument was carried out in triplicate, and the peak area of each tentative compound was measured in the obtained chromatogram for each replicate. After the selection of an adequate commercial standard, based on structural similarity with the target analyte, the concentrations of the identified phenolic compounds were calculated by the interpolation of the peak area detected in the replicate analysis of each sample in the corresponding surrogate standard calibration curve. The phenolic content was expressed as mean concentration ± standard deviation for each sample.

### 2.9. Statistical Analyses

The experiments were performed in triplicate and comparisons were made using the SPSS statistical software (SPSS version 28; SPSS Inc., Chicago, IL, USA). Analysis of variance (ANOVA) and Tukey’s post hoc tests with α at 0.05 were applied to determine statistical differences among conditions and digestive phases at a 95% confidence level.

## 3. Results and Discussion

### 3.1. Characterization of Olive Leaf Extract

In the present study, a commercial olive leaf extract was used to assess its stability along the gastrointestinal digestion process and the influence of its co-administration with inulin and microencapsulation. The extract contains, according to the manufacturer’s specifications, oleuropein (OLE) and hydroxytyrosol (HT) in contents around 48% and 1%.

**Figure 1:**
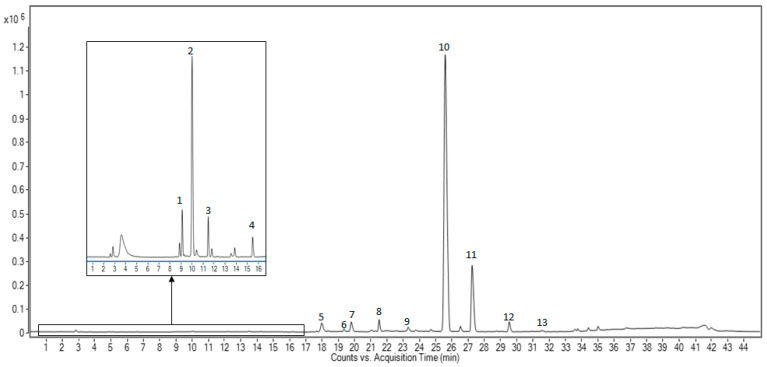
Base peak chromatogram of olive leaf extract at a concentration of 500 mg/mL, where the peaks have been numbered according to their elution order.

This extract was comprehensively characterized using HPLC-MS by using the method previously described and the obtained chromatogram is shown in Figure 1. Major and minor compounds detected were tentatively identified by the interpretation of their MS spectra obtained using a TOF-MS combined with the data provided by databases and the literature. These compounds were also quantified by using surrogate standard approximation, as described in Section 2.8. The composition of the commercial extract is summarized in Table 1.

### 3.2. Microencapsulation of Olive Leaf Extract by Spray-Drying

With the purpose of achieving an optimization of the encapsulation process for HT and OLE contained in the commercial olive leaf extract, several statistical analyses were conducted. For all evaluated responses, model adequacy, lack-of-fit, R^2^, and ANOVA were performed to determine the fitting of the proposed experimental model. In this sense, model adequacy was used to indicate the best-choice mathematical model; the lack-of-fit test revealed the fitting quality of the model applied; R^2^ revealed the ability to predict the behavior of the response variables and, finally, ANOVA indicated the statistically significant effect of temperature (X_1_) and the extract:encapsulating agent (E:EA) ratio (X_2_) on the response variables [45].

The experimental conditions of the different runs of the design performed to optimize the encapsulation together with the results of the encapsulation degree of HT and OLE are shown in Table 2. The analysis of variance (ANOVA) of the proposed experimental model for each response variable (%EE of HT and OLE) is summarized in Table 3.

As can be observed in Table 2, the encapsulation rate of HT ranged from 5.24% (run 9) to 82% (run 10). Concerning the fitting parameters (Table 3), the model adequacy was revealed to be satisfactory (*p* ≤ 0.05), lack-of-fit was *p* > 0.05, and R^2^ was 0.98, indicating the good fitting and predictive capabilities of the proposed model. Moreover, the ANOVA results were used to discern the effect of each factor on the encapsulation of HT. In this sense, the linear and quadratic effects of the E:EA ratio ($X_{2}$) were the most influential factors on the encapsulation degree of HT. Thus, a simplified equation that explains the behavior of this response is displayed in Equation (5):

(5) $$\%\mathrm{EE}\;\mathrm{HT}=-38.57+60.16X_{2}-7.51X_{2}^{2}$$

**Figure 2:**
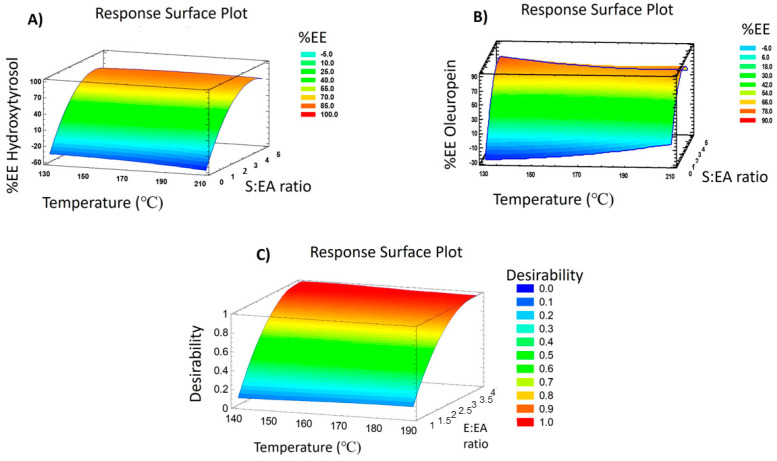
Response surface plots of (**A**) %EE of HT, (**B**) %EE of OLE, and (**C**) %EE of HT and OLE maximized simultaneously.

A graphical explanation of the proposed equation can be observed in Figure 2A, which helps to explain the results obtained for HT after performing the experimental design. It is possible to observe the relevant positive effect of the E:EA ratio, reaching a higher degree of encapsulation with higher inulin concentration independently of the effect of temperature, since it did not significantly influence this response. The higher availability of the encapsulating agent when a higher inulin concentration was applied, facilitated the entrapment of HT, increasing the encapsulation efficiency of this compound [49]. Therefore, the optimum conditions provided by the model for maximum HT encapsulation were 160 °C and an E:EA ratio of 4.00. When these conditions were applied, the predicted theoretical value for the response variable and the experimental result were quite similar (82.50% and 84.50%, respectively).

On the other hand, after performing the proposed experimental design, the encapsulation efficiency reached for OLE ranged from 13.72% (run 6) to 72.75% (run 11) (Table 2). Regarding the statistical analysis (Table 3), the model adequacy was *p* ≤ 0.05, the lack-of fit test was *p* > 0.05, and R^2^ was 0.93, revealing a good adjustment of the model as well as a good predictive capacity. Similar to the results found for HT, the linear and quadratic effects of the E:EA ratio ($X_{2}$) were shown to be also determinant in the OLE encapsulation efficiency, having positive effects on the encapsulation degree (Equation (6)).

(6) $$\%\mathrm{EE}\;\mathrm{OLE}=-19.84+48.44X_{2}-6.36X_{2}^{2}$$

The behavior of this response throughout the experimental design is shown in Figure 2B. The response surface plot for OLE encapsulation displays the positive effect of the concentration of inulin in the mixture introduced in the spray-drying to achieve a higher encapsulation of this compound. In spite of not having statistically significant effects, the temperature seemed to cause a slight decrease in the encapsulation efficiency of OLE. This result can be associated with the degradation of OLE at higher temperatures and, also, with the glass transition temperature of inulin. Thus, when the temperature is below this point the physical properties of the polymer change to those characteristic of the glassy or crystalline state. Hence, temperatures above the glass transition temperature cause a sticky behavior of the polysaccharide, hindering the recovery of inulin microparticles [50]. For these reasons, the proposed conditions to maximize OLE encapsulation were 135 °C and an E:EA ratio of 4.05, reaching a theoretical value for %EE of OLE of 77.20%. After applying the proposed optimum conditions, the veracity and the ability to predict the behavior of this response by the model were confirmed, since the experimental value was 82.13%. These results were similar to those obtained by Gonzalez et al., but with a much higher total OLE content (111 mg OLE/g of microparticles vs. 24.5 mg OLE/g of microparticles) [37].

At this point, it was decided to simultaneously optimize both response variables. For this purpose, the desirability function was used to find those conditions that provided predictable and reliable information. Therefore, the variable responses %EE of HT and OLE were maximized simultaneously and are plotted in Figure 2C considering their performance as a function of the two factors assessed.

The highest desirability index (value DI = 1) was found at 145 °C and an E:EA ratio of 3.87, obtaining theoretical values of 82.04% and 74.56% for %EE HT and %EE OLE, respectively. After performing experiments with the proposed optimum conditions identified through the desirability function, the experimental results (%EE HT 80.44% and %EE OLE 79.45%) were similar to those predicted (Table 4). Moreover, the total amounts of HT (2.3 mg/g of microparticles) and OLE (93 mg/g of microparticles) were obtained using HPLC-MS in order to evaluate the final concentration after the spray-drying process. Finally, in order to obtain complete information on the degree of encapsulation of minor compounds contained in the optimized microencapsulated powder, an exhaustive study of the degree of encapsulation of the remaining compounds was carried out. In addition, these results will help to understand in more detail the protection and controlled release of other olive bioactive compounds after in vitro digestive processes carried out in the subsequent trials. Overall, the results displayed in Table 4 reveal a high encapsulation degree of phenolic alcohols, secoiridoids, and flavonoids, with hydroxytyrosol and its glycoside being the most encapsulated compounds (80.44% and 79.91%, respectively). On the other hand, secoiridoids presented encapsulation degrees from 58.70% to 79.45%, with oleuropein being the most encapsulated in this group, followed by oleoside/secologanoside isomer 1 (76.96%). It should be noted that the differences in the degree of encapsulation of the different isomers may be due to conformational differences of the compounds, causing steric hindrance due to the prevented exposure of the functional groups that interact with the encapsulating agent, and consequently, reducing the interaction capacity [51,52]. Flavonoids also presented a great encapsulation degree (above 70%). This results may be associated with the interaction between the hydroxyl groups of the flavonoids and inulin, promoting the interaction between hydrogen bonds and, hence, increasing the encapsulation efficiency of these compounds [53]. Additionally, verbascoside, a phenylpropanoid, also presented a high encapsulation degree of 73.94%.

Thus, the proposed optimized conditions will allow for the optimal encapsulation of the leaf extract for HT and OLE, independent of their initial contents, while maintaining an adequate encapsulation efficiency of the other phenolic compounds present.

### 3.3. Influence of the Digestive Simulation of the Phenolic Profile

After the optimization of the encapsulation, the impact of this formulation on the stability under gastrointestinal conditions of the phenolic-rich extract was evaluated through the in vitro digestion of the three formulations consisting in the free extract, its co-administration with inulin, and the microencapsulated formulation.

#### 3.3.1. In Vitro Digestion of the Phenolic-Rich Extract

**Figure 3:**
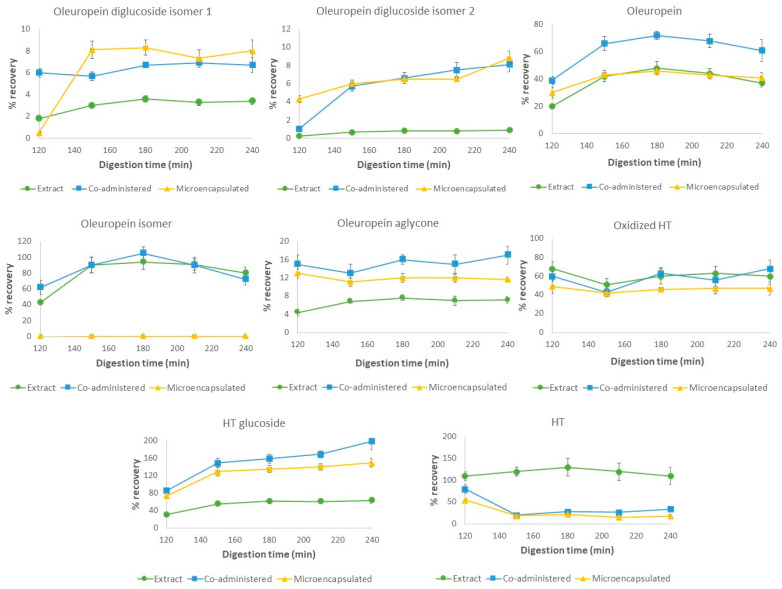
Evolution of oleuropein diglucoside isomers, oleuropein, oleuropein aglycone, and hydroxytyrosol and its oxidized and glucoside forms under gastric (120 min) and intestinal (150, 180, 210 and 240 min) stages of in vitro gastrointestinal digestion.

**Figure 4:**
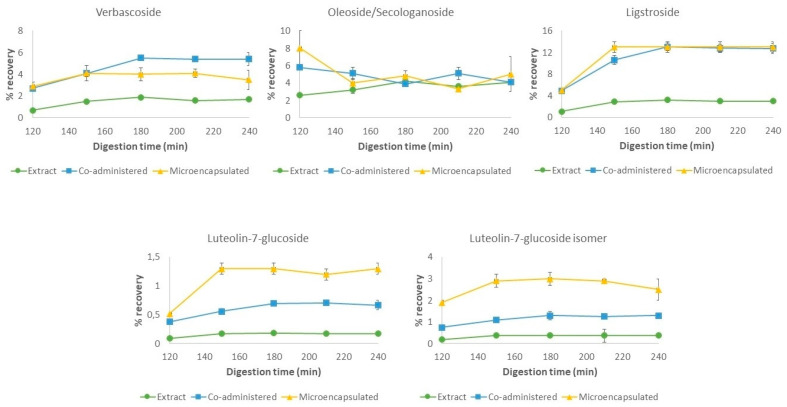
Evolution of verbascoside, oleoside/secologanoside, ligstroside, and luteolin-7-glucoside and its isomer under gastric (120 min) and intestinal (150, 180, 210 and 240 min) stages of in vitro gastrointestinal digestion.

Firstly, the simulated gastrointestinal digestion of the non-encapsulated extract was assayed, with the evolution of the recovery of the main phenolic compounds in the bioaccessible fractions shown in Figure 3 and Figure 4. Most of the quantified compounds seem to present a similar behavior under gastrointestinal digestion, with the the presence of oleoside/secologanoside isomers, verbascoside, luteolin-7-*O*-glucoside, ligstroside, oleuropein diglucoside, and its aglycone being reduced. On the other hand, HT, its glucoside and oxidized forms, and OLE were present in high percentages in the gastric phase.

At the end of the gastric step, there was a decrease in the phenolic content in the bioaccessible fraction, while high values were present in the residual fraction (data shown in Supplementary Material). As phenolic compounds present a poor stability under taxing environmental conditions, including the gastric low pH, this behavior appears to be related to a combined effect of both degradation and low solubility under these conditions. Both facts may render the recovery of the bioaccessible fraction significantly lower than in the rest of the digestive process. As previously reported, gastric degradation of some phenolic compounds, such as OLE, seems to be acid-catalyzed [54,55,56,57,58]. This is enhanced by the administration under fasting conditions, as intragastric pH values are significantly lower than under a fed state [33,59]. Thus, the high recovery values of HT under gastric conditions could be a result of OLE acidic degradation, as has been previously described in the literature [54,55,60,61]. Additionally, the impact of interactions with pepsin on bioaccessibility could also be considered. However, although some interactions have been described, more evidence is still needed to correctly assess their impact on phenolic digestion dynamics [62,63].

During the intestinal stage, all secoiridoids presented an increase in content in the bioaccessible fraction, which was more significant for OLE and HT glucoside, while HT remained stable, with a recovery above 100%. The observed decrease in residual content may relate this phenomenon to an improvement in the solubility of these compounds under intestinal conditions, related to the nature of the simulated fluids. Additionally, the neutral nature of the intestinal pH conditions could also imply less acidic degradation during this stage compared to the gastric conditions, as has been previously reported [31,56,57,58]. Indeed, a slower degradation of oleuropein has been previously described under pH values around 4–6 when compared to pH 2 while maintaining the same temperature [54].

Therefore, although an increase in the solubility, maintaining stable recovery percentages during the intestinal phase, has been observed, the aggressive effect of gastric conditions on phenolic stability results in the degradation and alteration of the initial content, which poses an obstacle for the phytochemicals’ survival in the gastrointestinal tract.

#### 3.3.2. Gastrointestinal Behavior of the Phenolic Co-Administration with Inulin

In this stage, as interactions of phenolic compounds with diverse carbohydrates are being widely studied, the impact of the non-encapsulated co-administration with inulin of the selected phenolic extract under in vitro gastrointestinal digestion was evaluated to assess its potential interaction. As presented in Figure 3 and Figure 4, the phenolic compounds presented higher recovery rates during the digestion, with a similar pattern to the one described in the previous section, as solubility seems to improve the recovery in the bioaccessible fraction. However, the behavior of HT and HT glucoside results in a very different profile to that depicted for the previous experiment.

When co-administered with inulin, the recovery of HT in the bioaccessible fraction was reduced from 80 to 20% when entering the intestinal phase, a contrasting result from the high recovery mentioned in the previous section for the free extract. On the other hand, the HT glucoside content in the bioaccessible fraction is increased by up to 200% at the end of the digestion, implying the degradation of its precursors, such as OLE diglucoside, into this compound. Additionally, the lesser degradation of all secoiridoid precursors to the non-glucosylated form, including HT glucoside, and a loss in soluble HT, could result in a lower bioaccessibility compared to the digested extract.

Thus, the combined administration of the polyphenolic-rich extract with inulin showed an increase in bioaccessibility and an alteration in the previously observed degradation profile, which could be related to a specific interaction between both components. Indeed, dietary fiber has been shown to have an effect on the release, digestion, bioaccessibility, and bioavailability of polyphenols through the gastrointestinal tract [64]. The interactions of polyphenols with macronutrients have gained interest in recent years, with a special focus on their interactions with carbohydrates and dietary fiber, such as inulin. Hydrogen bonds, electrostatic attraction, van der Waals forces, hydrophobic interactions, esterification, or physicochemical entrapment are the primary mechanisms underlying the interactions between these compounds [65,66,67]. The type and strength of the observed interactions depend on several endogenous and exogenous factors, including the polyphenol structure and functional groups due to methylation, methoxylation, and glycosylation [68]. However, there is a lack of consistency on the reported effect of the co-administration with different phenolic sources, as both an increase and decrease in the bioavailability of phenolic compounds have been observed when administered with dietary fiber [69,70].

Our results show a protective effect that increases the bioaccessibility of these compounds at the end of the small intestine digestion, which is in line with Guimarães et al., 2020, where an increase in the phenolic content along the gastrointestinal process was observed [71]. Similar results were presented in Vidal-Fonteles et al., 2021, where bioaccessibility was significantly higher at the end of the digestive process as compared with the non-thermally treated control [72]. However, in Tomas et al., 2018, a reduction in these parameters was observed for a tomato sauce manufactured with inulin [73]. This effect was explained through the development of hydrogen bonding between polyphenols and inulin, rendering it not available for absorption in the small intestine. Additionally, despite not completely protecting it from degradation, an improvement in stability was observed in an olive leaf extract introduced in a cereal-based food when compared to a fasting state [31].

In the present case, the interactions with inulin may be of a weaker nature as compared to those reported in the literature. However, the nature of the selected matrix for each study may have an impact on the observed results, as interaction with other components present in more complex food matrices such as sauces may influence the observed bioaccessibility. Additionally, an increased previous exposure of the phenolic compounds to the carbohydrate can also be observed, as in Tomas et al., 2018, when it was included during processing, prior to a homogenization process, while in this study it was directly incorporated into the co-digestion [73]. It has been reported that different technological processes, such as ultrasound treatment, may help accelerate the interaction between the compounds, establishing stronger bonds [74]. The reduced exposure of the extract and inulin source, as well as the lack of an accelerating process, may have restricted this interaction to weaker bonds, which could have been hydrophobic in nature (or similar), which were broken once the phenolic extraction process was achieved during the sample treatment with polar solvents, allowing the liberation of the phenolic compounds.

Moreover, the different nature of the phenolic compounds found in each source may also be considered, as the fiber–phenolic interaction seems to be also dependent on the nature of the structure of the specific compound, which may result in a different protection of some compounds compared to others [66]. This fact may be supported by the presented data, in which a higher affinity of HT and inulin may be the cause of the observed bioaccessibility, whereas this interaction may not be theorized for other of the identified compounds in the olive extract.

Overall, co-administration with inulin has been proved to have a protective effect on phenolic stability, as can be observed, firstly, in the higher bioaccessibility obtained for the bioactive compounds and secondly, in the alteration of the previously described degradation pattern found for some compounds, such as HT glucoside.

#### 3.3.3. Encapsulated Formulation

Finally, the effect of microencapsulation with inulin by spray-drying on the phenolic gastrointestinal behavior was evaluated. In this case, the observed phenolic profile progressed with a similar trend as the previous co-administration with the encapsulating agent. Under gastric conditions, a high percentage of the evaluated compounds was preserved in the encapsulated formulation, although higher recovery values were found for HT, and its glucoside and oxidized forms, as well as for the OLE isomer. As the digestive process continued, even though an increase in free phenolic content was observed, the data showed no significant differences along the intestinal phase, or only slight modifications with respect to the initial content in this phase.

In order to assess the evolution of the microencapsulated phenolic compounds, first we need to consider the encapsulation efficiency for each compound. As presented in Table 4, the encapsulation efficiency for all identified compounds tends to be high, reaching values between 70 and 80%. In this sense, although encapsulation was successful, a medium percentage of 20–30% of the initially presented compounds is not encapsulated and, therefore, remains as superficial content. This superficial polyphenol concentration is not protected by the inulin microcapsule and may be bioaccessible from the beginning of the digestion. In fact, the recovery in the bioaccessible fraction tends to be lower than the non-encapsulated percentage for each compound. This phenolic content may be liberated into the digestion medium by diffusion, where it could be degraded or may interact with the superficial inulin, as similarities can be found with the co-administration of the non-encapsulated formulation (which may later have an influence on its stability). This could be interesting because it would allow for a similar or higher bioaccessibility of these superficial compounds compared to the free extract, while ensuring the protection of the encapsulated phenolic content from digestion conditions along the upper gastrointestinal tract.

However, the presence of HT, its glucoside form, and the OLE isomer in the bioaccessible fraction does not correlate with their encapsulation efficiency and, thus, the already superficially available phenolic content. Specifically, recovery of HT glucoside is doubled by the end of the intestinal phase, showing similarities to the non-encapsulated formulation. In this case, the observed data may be attributed to the combined influence of their superficial content, degradation of liberated precursors, and the impact of the nature of the digestive conditions on microparticle stability (implying solubility, low pH, high time exposure, and high temperatures). The presence of inulin, as previously described, may favor the degradation of the liberated precursors into an HT glucoside.

With inulin being a non-digestible carbohydrate, no specific enzymes for its degradation are present in the upper gastrointestinal tract. However, this compound is still susceptible to acidic hydrolysis, specifically in the gastric step where critical parameters for this phenomenon such as low pH values, residence time, and temperature are present [75,76]. In this sense, the degradation rate varies depending on the structural characteristics of inulin and the properties of the glycosidic bond, as β-(2-1) bonds between the fructose units are susceptible to hydrolyzation under an acidic environment [77]. In fact, in Gonzalez et al., 2020 similar results were observed, where gastric hydrolysis of inulin in small amounts inducing the liberation of phenolic compounds from encapsulated formulations was also described [37].

Thus, slight acidic hydrolysis of inulin may have induced the liberation of phenolic compounds which, under gastric conditions, could be degraded into HT and HT glucoside. The increase in the intestinal stage may be attributed to a higher solubility under these conditions as previously mentioned. Nevertheless, these favorable conditions, especially a more neutral pH, seem to decrease this hydrolysis, as the content remains stable for the duration of the digestion.

The presented results exhibit the significant effect of the encapsulation process on the phenolic compounds’ stability and survival along the upper gastrointestinal tract. Although some bioaccessibility can be observed, it appears to be mainly attributed to the diffusion of their superficial content. This could also be related to a slight hydrolysis of inulin during the gastric phase. The described behavior differs from the free extract, with a stabilization through the rest of the digestion, which could be explained through a protective interaction mediated by the encapsulating agent. Additionally, although bioaccessible, the use of inulin as an encapsulation agent could further enhance the stability of the liberated compounds, rendering them stable through the process and accessible for later stages, their early colonic release, and use by colonic microbiota.

#### 3.3.4. Effect of the Encapsulation on the Digestive Phenolic Profile

Overall, a different phenolic profile can be observed for each of the studied administrations, with several similarities that are mainly found during the intestinal phase.

Firstly, the increased overall bioaccessibility and different phenolic profile of the co-administered formulation with regards to the digested extract may indicate that the presence of inulin appears to induce a protective effect through establishing moderately strong bonds. In fact, this behavior is also present in the digested microcapsules, suggesting a significant effect in this scenario of the encapsulated inulin on the liberated superficial compounds. However, this effect could be dependent on the phenolic structure and its interaction with the carbohydrate, modifying the degradation profile.

As previously stated, the microencapsulated extract exhibits less variability in recovery during the whole digestion process, with a high stability under intestinal conditions. Degradation and dynamism in the polyphenol content is increased in the non-encapsulated co-administration with inulin and even greater in the free extract. Indeed, although low bioaccessibility is desired and observed in the encapsulated formulation, the bioaccessibility values at the end of the intestinal phase highlight not only the viability of inulin as a protective agent, but also the consideration of this co-administration as a viable option for increasing phenolic survival under gastrointestinal conditions.

However, several similarities between the conditions have been observed, mainly focused on the intestinal phase. The presented results showed that co-administration with inulin exhibits an intermediate effect between the free and encapsulated extract. Nevertheless, the combined effect of the encapsulation and inulin protection may be presented as a more efficient strategy for gastrointestinal administration of a phenolic-rich extract.

In this sense, the previous literature on inulin’s interaction with bioactive compounds in vivo seems to support its protective effect and potentiality as an encapsulating agent. The administration of soybean isoflavones with and without the presence of inulin has been evaluated for their bioavailability in plasma in postmenopausal women [78]. The plasma concentrations of both daidzein and genistein were higher in the formulation with inulin than in the absence of this non-digestible carbohydrate. Similar effects were observed in the bioavailability of phenolic compounds from grapes co-administered with dietary fiber, where the total antioxidant capacity of these compounds in blood samples of healthy volunteers was increased when compared to the control group [79]. Additionally, the presence of pectin has been reported to increase the bioavailability of quercetin and isorhamnetin in mice compared to other formulations [80].

The presented in vitro results are in line with this preliminary in vivo evidence, and will further improve the development and evaluation of innovative approaches of the administration of phenolic compounds in protective formulations, enhancing their implementation into functional foods and nutraceutical formulations. This will provide the foundation for the future in vivo evaluation of promising formulations and new products for improving and maximizing their health benefits.

## 4. Conclusions

The presence of inulin and the microencapsulation of the extract during in vitro gastrointestinal digestion of the different formulations showed an alteration in the phenolic degradation profile. Indeed, the presence of inulin proved to have a beneficial effect on the phenolic stability, as interactions between inulin and the present phenolic compounds improved their protection under digestive conditions. Thus, the presence of inulin improves the gastric and intestinal stability of OLE and modulates the degradation of HT precursors, favoring the stability of its glucoside form. However, the combined effect with the encapsulation process could be a more attractive alternative as it would allow for an increased protection of the encapsulated compounds, although a slight degradation could be observed under gastric conditions, while maintaining the stability of the superficial (non-encapsulated) or liberated content. These presented results could contribute to the development of innovative formulations for obtaining functional foods and neutraceutical products with improved stability compared to the free extracts, thus maximizing the potential health benefits observed in their administration. Nevertheless, further research into inulin–phenol interactions during digestion is needed in order to fully elucidate its digestion dynamic.

## Figures and Tables

**Table 1:** Composition of the commercial olive leaf extract.

Peak	RT (min)	Proposed Compound	Molecular Formula	*m*/*z*	Concentration (%)
1	9.08	Hydroxytyrosol glucoside	C_14_H_20_O_8_	315.1108	0.107 ± 0.008
2	10.01	Hydroxytyrosol	C_8_H_10_O_3_	153.059	1.0 ± 0.1
3	11.61	Oleoside/Secologanoside isomer 1	C_16_H_22_O_11_	389.1135	0.26 ± 0.03
4	15.51	Oleoside/Secologanoside isomer 2	C_16_H_22_O_11_	389.1135	0.10 ± 0.02
5	18.00	Verbascoside	C_29_H_36_O_15_	623.199	0.67 ± 0.06
6	19.37	Luteolin-7-*O*-glucoside	C_21_H_20_O_11_	447.095	1.5 ± 0.1
7	19.81	Oleuropein diglucoside isomer 1	C_31_H_42_O_18_	701.231	0.38 ± 0.04
8	21.68	Oleuropein diglucoside isomer 2	C_31_H_42_O_18_	701.231	0.78 ± 0.01
9	23.19	Luteolin glucoside isomer	C_21_H_20_O_11_	447.095	0.28 ± 0.03
10	25.72	Oleuropein	C_25_H_32_O_13_	539.181	48 ± 1
11	27.24	Oleuropein isomer	C_25_H_32_O_13_	539.181	4.9 ± 0.2
12	29.57	Ligstroside	C_25_H_32_O_12_	523.182	0.89 ± 0.04
13	31.75	Oleuropein aglycone	C_19_H_22_O_8_	377.1223	0.24 ± 0.02

**Table 2:** Experimental design conditions with experimental and fitted results for the response variables (encapsulation efficiency of main compounds).

Run	T (°C)	E:EA	%EE HT		%EE OLE	
			Exp.	Pred.	Exp.	Pred.
1	195.25	2.50	66.68	62.17	64.96	64.33
2	134.75	2.50	69.82	65.77	67.62	61.99
3	165.00	2.50	60.73	65.49	72.27	60.49
4	165.00	0.69	8.15	5.28	13.73	9.31
5	190.00	4.00	80.35	81.11	70.23	70.22
6	140.00	1.00	10.77	16.27	13.72	18.31
7	165.00	2.50	67.12	65.49	49.10	60.49
8	165.00	2.50	65.21	65.49	53.08	60.49
9	190.00	1.00	5.24	9.02	26.18	27.74
10	165.00	4.32	81.89	81.77	71.62	69.78
11	140.00	4.00	81.33	81.81	72.75	75.77
12	165.00	2.50	64.32	65.49	64.14	60.49

T: inlet air temperature; E:EA: extract:inulin ratio; %EE: encapsulation efficiency; results are expressed as %; HT: hydroxytyrosol; OLE: oleuropein; Exp.: experimental; Pred.: predicted.

**Table 3:** Analysis of variance (ANOVA) of the proposed experimental model for each response variable.

Source	Encapsulation Efficiency	
	HT	OLE
	*p*-Value	*p*-Value
Model	0.000 ^a^	0.002 ^a^
X_1_: Temperature	0.2398	0.824
X_2_: E:EA ratio	0.000 ^a^	0.008 ^a^
X_1_ X_2_	0.482	0.744
$X_{1}^{2}$	0.458	0.529
$X_{2}^{2}$	0.00 ^a^	0.068 ^a^
Lack-of-fit	0.058	0.844
R^2^	0.98	0.93

E:EA: extract:encapsulating agent; R^2^: quadratic correlation coefficient; HT: hydroxytyrosol; OLE: oleuropein; ^a^ significant (*p* < 0.050).

**Table 4:** Encapsulation efficiency of phytochemicals found in the optimized microparticles powder.

Compound	Encapsulation Efficiency (%)
Hydroxytyrosol glucoside	79.91
Hydroxytyrosol	80.44
Oleoside/Secologanoside isomer 1	76.96
Oleoside/Secologanoside isomer 2	58.70
Verbascoside	73.94
Luteolin-7-O-glucoside	72.14
Oleuropein diglucoside isomer 1	70.88
Oleuropein diglucoside isomer 2	72.94
Luteolin glucoside isomer	70.78
Oleuropein	79.45
Oleuropein isomer	67.38
Ligstroside	66.40
Oleuropein aglycone	70.30

## Data Availability

All the data generated by this research have been included in the article. For any assistance, it is possible to contact with the corresponding authors.

## Supplementary Materials

The following supporting information can be downloaded at: https://www.mdpi.com/article/10.3390/nu16010093/s1, Figure S1: Evolution of the residual fraction of oleuropein diglucoside isomers, oleuropein, oleuropein aglycone, hydroxytyrosol and its oxidized and glucoside forms under gastric (120 min) and intestinal (150, 180, 210 and 240 min) stages of in vitro gastrointestinal digestion; Figure S2: Evolution of the residual fraction of verbascoside, oleoside/secologanoside, ligstroside and luteolin-7-glucoside and its isomer under gastric (120 min) and intestinal (150, 180, 210 and 240 min) stages of in vitro gastrointestinal digestion.
