# Supplementary material for: Evaluation of Olive Leaf Phenolic Compounds’ Gastrointestinal Stability Based on Co-Administration and Microencapsulation with Non-Digestible Carbohydrates
Source: Nutrients. 2023 Dec 27;16(1):93. doi: 10.3390/nu16010093 (PMC10780473; doi:10.3390/nu16010093)
Supplement: Supplementary file 1 [file nutrients-16-00093-s001.zip › nutrients-2725175-supplementary.pdf]

Supplementary material

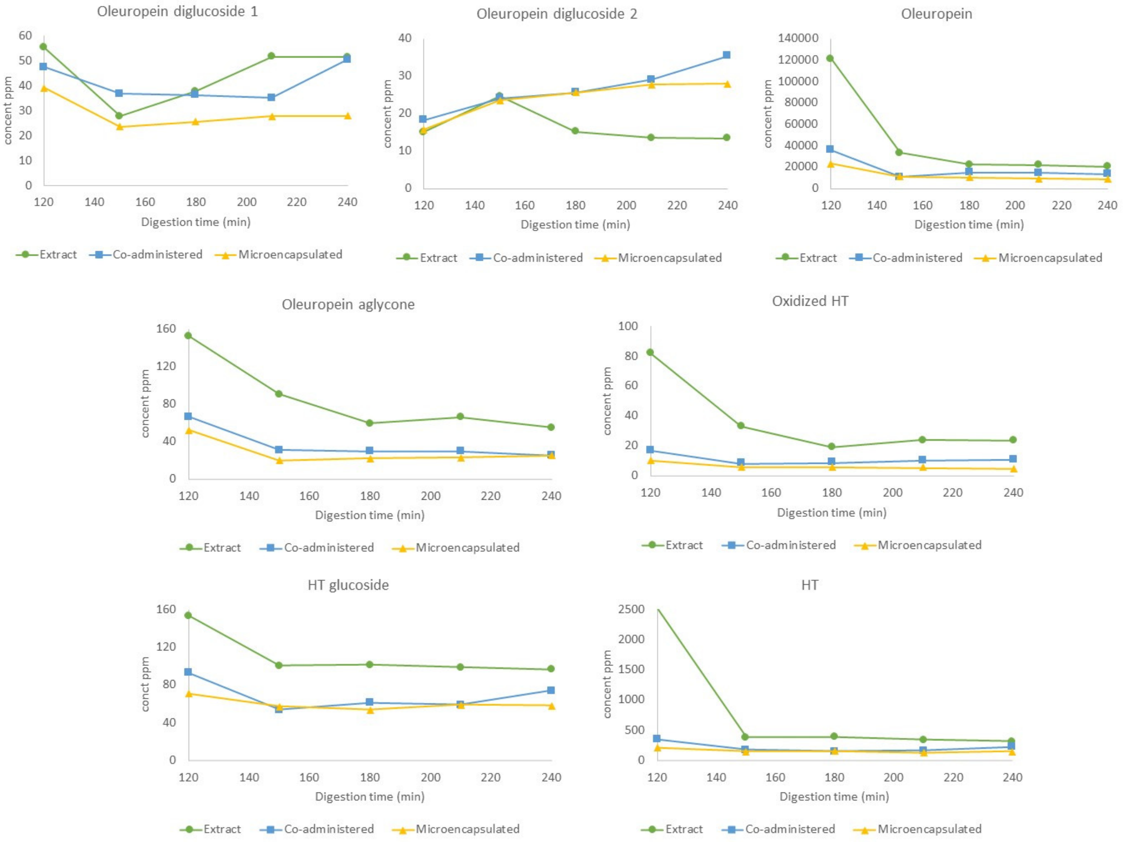

Figure S1. Evolution on the residual fraction of oleuropein diglucoside isomers, oleuropein, oleuropein aglycone, hydroxytyrosol and its oxidized and glucoside forms under gastric (120 min) and intestinal (150, 180, 210 and 240 min) stages of *in vitro* gastrointestinal digestion.

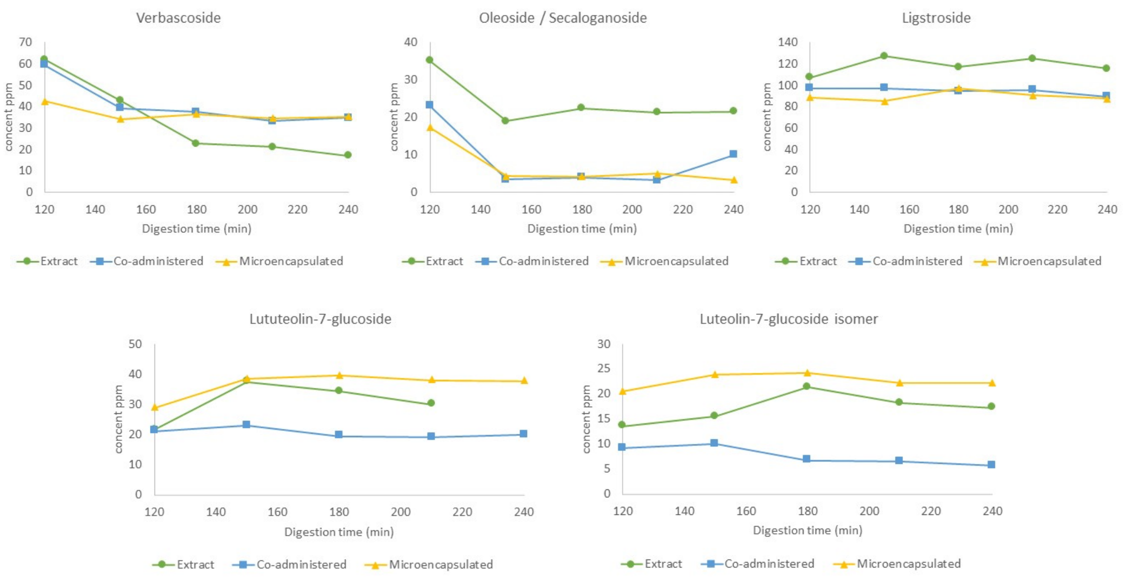

Figure S2. Evolution on the residual fraction of verbascoside, oleoside/secologanoside, ligstroside and luteolin-7-glucoside and its isomer under gastric (120 min) and intestinal (150, 180, 210 and 240 min) stages of *in vitro* gastrointestinal digestion.
